# Childhood Maltreatment Is an Independent Risk Factor for Prediabetic Disturbances in Glucose Regulation

**DOI:** 10.3389/fendo.2017.00151

**Published:** 2017-06-30

**Authors:** Li Li, W. Timothy Garvey, Barbara A. Gower

**Affiliations:** ^1^Department of Psychiatry and Behavioral Neurobiology, University of Alabama at Birmingham, Birmingham, AL, United States; ^2^Department of Nutrition Sciences, University of Alabama at Birmingham, Birmingham Veterans Affairs Medical Center, Birmingham, AL, United States; ^3^Department of Nutrition Sciences, University of Alabama at Birmingham, Birmingham, AL, United States

**Keywords:** childhood maltreatment, insulin sensitivity, glucose intolerance, diabetes risk, beta cell function, prediabetic state

## Abstract

**Aims:**

Childhood maltreatment (CM) is shown to be associated with obesity and depression. However, the relationship of CM to prediabetic state is much less studied. We tested the hypothesis that CM increases the risk for prediabetic state due to glucose intolerance, reduced insulin sensitivity, and beta cell function.

**Methods:**

Oral glucose tolerance test (OGTT)-derived metabolic parameters of glucose tolerance, insulin sensitivity, and beta cell function were measured in 121 participants aged 19–60 years. CM exposure was measured using the Childhood Trauma Questionnaire. Blood samples were collected to measure the inflammatory factors.

**Results:**

After controlling for age, race, gender, education, and depression, about 15% higher glucose area under the OGTT curve was observed in the CM group. CM individuals also exhibited impaired insulin sensitivity manifested by the Matsuda index and homeostasis model assessment of insulin resistance, which were correlated with CM severity after adjusting for depression. CM group showed approximately 50% lower disposition index. C-reactive protein and tumor necrosis factor-α levels were greater in the CM group vs. the non-CM group, and both were correlated with CM severity (*r* = 0.21, 0.23, respectively, both *p* < 0.05). Multiple regression analyses revealed that CM contributed to reduced insulin sensitivity and lower disposition index independent of depression and visceral fat mass.

**Conclusion:**

These data suggest an important relationship between CM and increased risk for prediabetic state due to glucose intolerance, impaired insulin sensitivity, and beta cell function. Our findings indicate that CM appears to be an independent risk factor for developing prediabetes.

## Introduction

Type 2 diabetes (T2D) is preceded by prediabetes, which is characterized by impaired fasting glucose levels and/or impaired glucose tolerance (1). Epidemiological studies showed that about one-third of prediabetic individuals develop T2D over a 5–10 year follow-up period (2). T2D is also characterized by defects in pancreatic beta cell function and insulin sensitivity, which have been demonstrated long before overt diabetes in individuals with impaired glucose tolerance (3–5). Thus, the use of impaired glucose tolerance and beta cell function as well as decreased insulin sensitivity as the predictors to identify individuals at high risk for T2D is important. Given the worldwide prevalence of T2D and its associated morbidity and mortality (6), it is critical to identify individuals at high risk for T2D in order to provide proper interventions to slow or even prevent progression to overt diabetes.

Childhood maltreatment (CM) presents a major public health concern. CM occurs at an epidemic rate, with nearly 700,000 substantiated reports each year in the U.S., though this is likely an underestimate of the true number (7, 8). Maltreatment occurring during childhood may have more deleterious consequences than in adulthood because childhood is a period of emotional, behavioral, and physical development. Epidemiological studies have clearly indicated that CM is a non-specific risk factor for various psychiatric syndromes (9–12), and poses an increased risk for obesity and cardiovascular diseases in adulthood, although the underlying mechanisms are not well defined (13–15). The estimated cost of CM-related diseases in the U.S. alone is over $124 billion per year (16). Therefore, understanding the association between CM and related chronic diseases, T2D in particular, in adulthood may aid in prevention and intervention efforts, ultimately reducing the high rates of associated mortality, morbidity, and medical cost.

CM may be one factor that contributes to impaired glucose tolerance and thereby inducing prediabetic state and the subsequent T2D. The possible connection between CM and prediabetic state is suggested by several avenues of research, and their relationship may be mediated *via* risk factors for T2D. For example, a recent meta-analysis found that CM was associated with a 40% increased risk of developing obesity in adulthood, a known risk factor for T2D (17, 18). Similarly, CM has been shown to predict unhealthy behaviors, including physical inactivity and cigarette smoking, in both adolescents and adults, which are also risk factors for T2D (19–22). In addition, depression, a common risk factor of T2D, is a major outcome of CM (9, 23–25). As such, these risk factors may be in the causal pathway between CM and prediabetic state. However, whether CM is independently associated with increased risk for prediabetic state is rarely studied. Compared with other well-established risk factors for prediabetic state, the role of psychosocial factors, CM in particular, in the development of glucose dysregulation has received much less attention. Although the importance of beta cell dysfunction and decreased insulin sensitivity in developing T2D is established, few studies have focused on the effects of CM on pancreatic beta cell function and insulin sensitivity in humans directly. Therefore, it is imperative to determine CM impacts on glucose metabolism, insulin sensitivity, and beta cell function.

Accumulating evidence has demonstrated that individuals with CM showed increased levels of C-reactive protein (CRP) and circulating cytokines, including tumor necrosis factor-α (TNFα) (26, 27). Individuals with obesity are prone to a chronic, low-grade inflammation as the metabolically active visceral fat produces pro-inflammatory cytokines (28). Similarly, T2D is also characterized by chronic, low-grade inflammation that may be a consequence of obesity, or a sequela of obesity-induced diabetes (17, 18). Taken together, low-grade inflammation may be a potential mechanism linking CM with the risk for prediabetic state and the subsequent development of diabetes.

To our knowledge, evidence is lacking to link prediabetic state in adulthood as a possible long-term consequence of CM. To address this issue, experiments were designed to examine whether CM is an independent risk factor for prediabetic state and its potential mechanisms. The primary objective of this study was to investigate the relationship between CM and oral glucose tolerance test (OGTT)-derived measures of glucose tolerance, insulin sensitivity, and beta cell function in adults with adverse childhood experiences. Furthermore, we explored whether any observed associations between CM and OGTT-derived measures were dependent on obesity and the inflammatory factors.

## Research Design and Methods

### Study Sample and Design

Participants were recruited through the University of Alabama at Birmingham hospital and the community in Birmingham, AL, USA. The project was approved by the Institutional Review Board at the University of Alabama at Birmingham. Each participant was consented and provided with a copy of written consent prior to completing any research procedures. One-hundred fifty subjects were enrolled, but only 121 participants completed the study, including males and females aged 19–60 years. The exclusion criteria were endocrine disorders (e.g., hypothyroidism, hyperthyroidism, and diabetes), use of systemic corticosteroids or drugs affecting glucose metabolism and pregnancy or lactation. The subjects self-reported their age, race, medical history, and level of education. A self-reported scale, Beck Depression Inventory (BDI), was used to assess depression symptoms in participants.

The subjects completed a modified version of a Childhood Trauma Questionnaire (CTQ), which is a well-validated measure of child abuse and neglect before the age of 18 (10–12, 29). The CTQ is comprised of 28 items on five subscales, including physical neglect (PN), emotional neglect (EN), emotional abuse (EA), physical abuse (PA), or sexual abuse (SA) (Table 1). Subjects rated statements about childhood experiences on a five-point scale (1 = “never true” to 5 = “very often true”). The CTQ score in each participant was calculated by adding the score for each subscale, which ranged between 5 and 25. The CTQ is repeatedly shown to be a reliable and valid measure of CM with both a clinician-rated interview of childhood abuse and therapists’ ratings of abuse (10–12). A cut-off score higher than 8 on the PN, SA, or PA, and cut-off score higher than 10 and 15 for EA or EN, respectively, have provided good sensitivity and specificity for confirmed abuse or neglect (10–12, 29). A subject was considered to have a history of CM if any of the subscale scored greater than stated above and/or CTQ score greater than 35 in this study. The sample was divided into CM (*n* = 69) or non-CM (*n* = 52) groups according to their CTQ measurement (Table 1).

**Table 1:** Participants characteristics.

	Non-CM (*n* = 52)	CM (*n* = 69)	*p* Values
Women/men, *N*	36/16	43/26	0.696
Age, years	35.0 ± 1.6	39.2 ± 1.6	0.052
Race, C/AA	21/31	35/34	0.248
Education, ≥10 years (%)	90.3	87.3	0.889
BMI, kg/m^2^	31.2 ± 0.96	29.9 ± 0.84	0.451
Waist-to-hip ratio	0.84 ± 0.01	0.86 ± 0.02	0.556
BDI	5.88 ± 1.43	12.56 ± 1.55	0.003
**Childhood maltreatment**			
PN	5.3 ± 0.1	8.6 ± 0.4	<0.001
PA	6.2 ± 0.2	9.8 ± 0.6	<0.001
EN	6.6 ± 0.2	14.8 ± 1.5	<0.001
EA	5.9 ± 0.2	11.9 ± 0.7	<0.001
SA	5.2 ± 0.1	9.4 ± 1.5	<0.001
Total	29.1 ± 0.4	52.6 ± 2.2	<0.001

*CM, childhood maltreatment; C, Caucasians; AA, African Americans; BMI, body mass index; BDI, Beck Depression Inventory; PN, physical neglect; PA, physical abuse; EN, emotional neglect, EA, emotional abuse; SA, sexual abuse*.

### Oral Glucose Tolerance Test

All participants underwent a standard 2-h OGTT after an overnight fast (10–12 h) and were administered a 75 g oral glucose solution (30). Blood samples were collected at 0, 0.5, 1, and 2-h post-glucose load for the determination of glucose and insulin levels. Glucose levels were measured using the glucose oxidase method with a glucose analyzer (Beckman Coulter Unicel DxC 800). Insulin was determined using an enzyme labeled chemiluminescence immunoassay (Siemens). Incremental area under the curve (AUC) for glucose response and insulin secretion during the 2-h OGTT was calculated using the trapezoidal method.

### Insulin Sensitivity and Beta Cell Function Assessment

The Matsuda index was utilized as a surrogate estimate of insulin sensitivity. The Matsuda index is a validated insulin sensitivity index that reflects an estimate of both hepatic and muscle insulin sensitivity (31). It is widely used for estimating insulin sensitivity since it is highly correlated with the gold standard—the hyperinsulinemic–euglycemic clamp method (31). It is calculated using fasting glucose and insulin data taken from the OGTT, and the mean data representing the average glucose and insulin levels obtained during the OGTT at the four time points as above. The homeostasis model assessment of insulin resistance (HOMA-IR) was calculated according to the formula: [fasting insulin (μU/L) × fasting glucose (nmol/L)]/22.5, as an additional index for insulin resistance (32).

Pancreatic beta cell function was assessed using OGTT-derived insulinogenic index and disposition index (DI_OGTT_). The insulinogenic index correlates well with insulin secretion measured by the hyperinsulinemic-euglycemic clamp and was calculated by the ratio of the incremental response of insulin to glucose at 30 min of the OGTT (33). DI_OGTT_ is a composite measure that captures both insulin secretion and insulin sensitivity, and it was calculated for this study as insulinogenic index × Matsuda index (34).

### Blood Sample Collection and Measurement

Blood samples (10 mL) were drawn from each participant by venipuncture. The blood was centrifuged at 3,000 g for 10 min that was immediately divided into aliquots and frozen at −80°C until samples were analyzed. Plasma concentrations of CRP (minimum sensitivity = 0.50 mg/L, intra- and inter-assay CV of 6.1 and 7.5%, respectively) were analyzed using immunoassay on a Stanbio Sirrus Analyzer. Plasma levels of TNFα (minimum sensitivity = 0.20 pg/mL, intra- and inter-assay CV of 4.2 and 5.6%, respectively) were analyzed using multiplex human immunoassay kits (Meso Scale Discovery, Gaithersburg, MD, USA). All samples were run in duplicate, and the mean of the duplicate samples was reported.

### Determination of Body Composition

Body weight and height were measured to calculate body mass index (BMI) using the Quetelet index (kilograms per square meter). Waist and hip circumferences were measured for the calculation of waist-to-hip ratio. A dual-energy X-ray absorptiometry (GE-Healthcare, Madison, WI, USA) scan was acquired with a total body scanner for each participant to determine visceral fat amount as previously described (35). In brief, participants were required to wear light clothes and lie supine with arms at their sides while undergoing the scan. They were not allowed to wear any metal objects. CoreScan software was used to estimate the visceral fat mass based on measurement of abdominal area and subcutaneous adipose tissue during the scan.

### Statistical Analysis

Data were analyzed using the Statistical Package for the Social Sciences version 23 (SPSS Inc., Chicago, IL, USA). Data were presented as mean ± SE in the text, figures, and tables. *p* Values ≤0.05 were considered significant. Normal distribution and homogeneity of variances were confirmed by Shapiro–Wilk’s and Levene’s tests, respectively. Before data analyses, insulin values, Matsuda index, insulinogenic index, DI_OGTT_, CRP, and TNFα were log transformed so that each of these variables followed an approximate normal distribution.

Differences between the CM and the non-CM groups in variables of interest were compared using Chi-square test for categorical data and analysis of covariance for continuous data with adjustment for age, race, gender, education, and depression. Partial correlation coefficients controlling for age, race, gender, education, and depression were also calculated to evaluate associations between CM scores and metabolic parameters. In addition, stepwise multiple regression analyses were used to determine which, if any, risk factors for prediabetic state were most predictive of glucose area under the OGTT curve, HOMA-IR, and beta cell function (i.e., DI_OGTT_).

## Results

### Participant Characteristics

Descriptive characteristics of the participants in the non-CM and the CM groups are presented in Table 1. Of the 121 participants, no significant differences were observed between groups in gender, race, education level, BMI, and waist-to-hip ratio. There was a trend toward greater age in the CM group (*p* = 0.052). As expected, the CM group was found to have greater subscale and total scores of CM. In addition, the CM group scored much higher regarding self-reported depression symptoms that was measured by the BDI.

### Glucose and Insulin Responses to the OGTT

**Figure 1:**
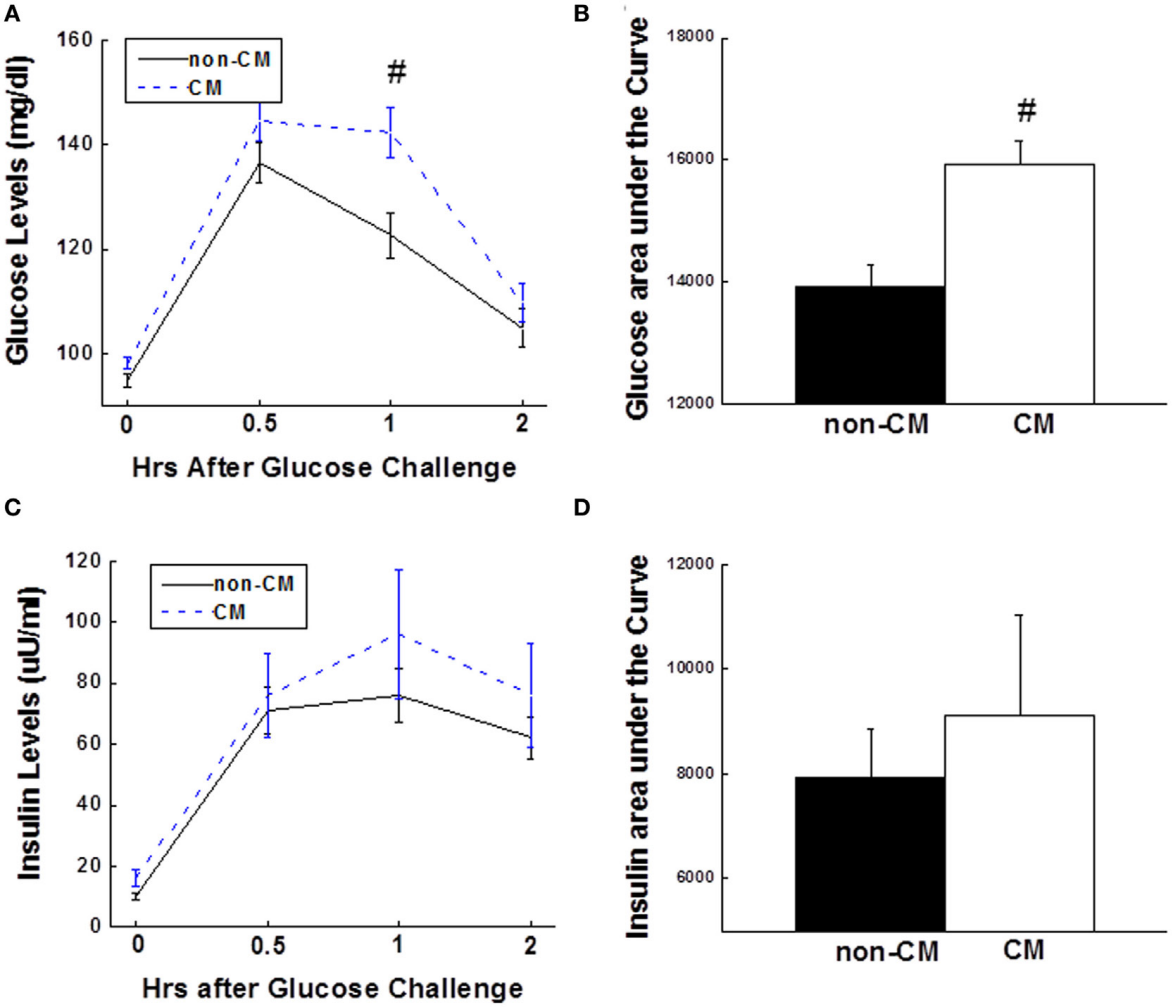
**(A)** 2-h plasma glucose levels during the OGTT. **(B)** Bar graph of glucose AUC during the OGTT. **(C)** 2-h insulin levels during the OGTT. **(D)** Bar graph of insulin AUC during the OGTT. Data are presented as the mean ± SE in each group. Dashed line stands for the CM group and solid line stands for the non-CM group; ^#^*p* < 0.01; compared with the non-CM group. CM, childhood maltreatment; OGTT, oral glucose tolerance test; AUC, area under the curve.

As shown in Figure 1A, fasting glucose did not differ significantly between the two groups; however, 1-h glucose levels were significantly greater in the CM than the non-CM group (Figure 1A). About 20% of the participants in the CM group had evidence of prediabetes (impaired fasting glucose and/or impaired glucose tolerance) as compared with 9.6% of the participants in the non-CM group (*p* < 0.01). Insulin levels at the four time points of the OGTT did not differ significantly between the two groups (Figure 1C). The CM group had significantly higher (~15%) glucose AUC (*p* = 0.008), but the insulin AUC was not different during the OGTT (*p* = 0.91), indicative of relative insulin deficiency in the CM group (Figures 1B,D).

### Relationship between CM and Indices of Insulin Sensitivity and Beta Cell Function

**Figure 2:**
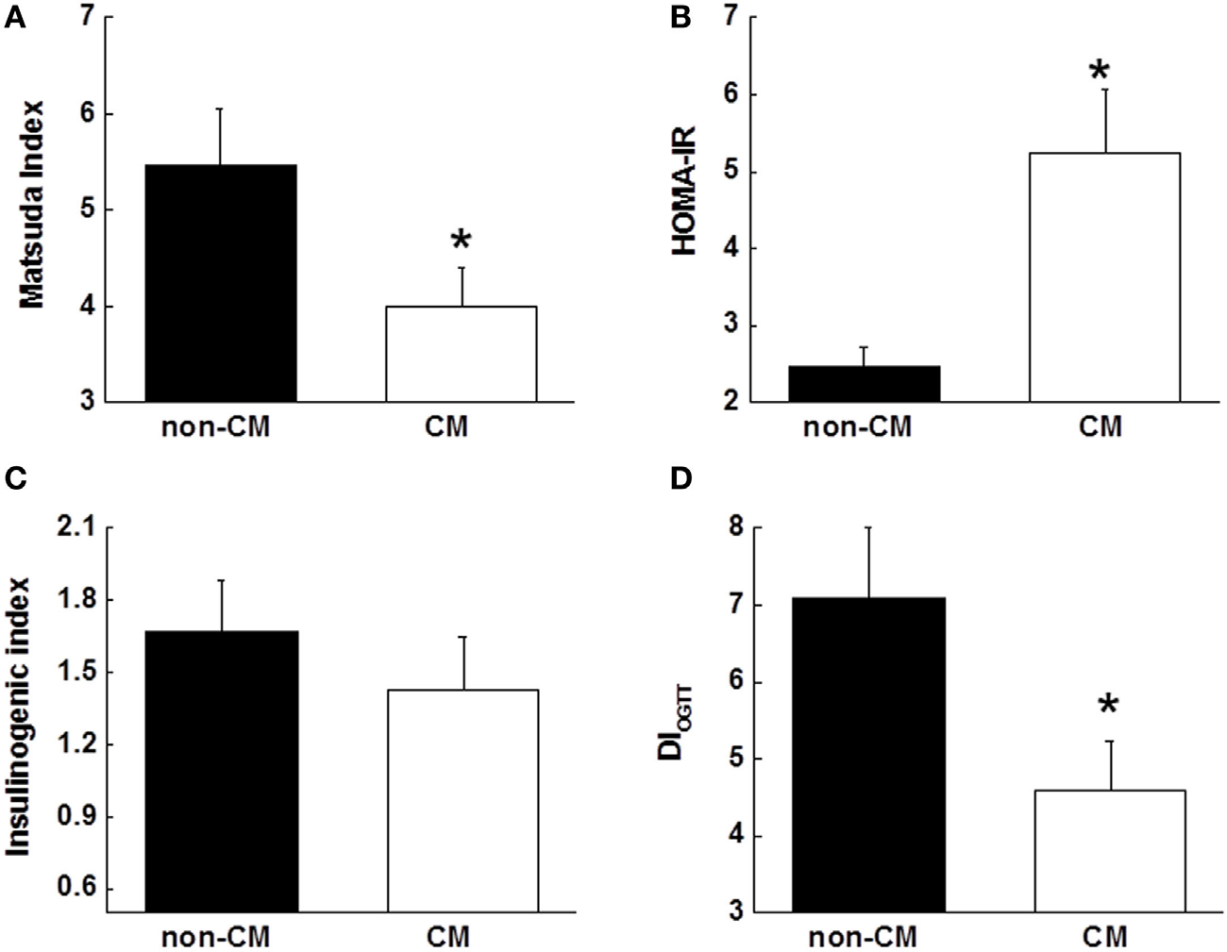
**(A,B)** Surrogate indices of insulin sensitivity, including Matsuda index, and HOMA-IR. **(C)** Insulin response to oral glucose challenge during the OGTT that was measured using insulinogenic index (InsAUC_30_/GluAUC_30_). **(D)** Disposition index that was measured using DI_OGTT_ (insulinogenic index × Matsuda index). Data are presented as the mean ± SE in each group. *p < 0.05; compared with the non-CM group. CM, childhood maltreatment; HOMA-IR, homeostasis model assessment of insulin resistance; OGTT, oral glucose tolerance test; DI_OGTT_, OGTT-derived disposition index.

Insulin sensitivity, as estimated by the Matsuda index and HOMA-IR, was significantly different in the CM group compared with the non-CM group (Figures 2A,B). The acute insulin response to glucose measured as the insulinogenic index during the OGTT was not significantly different between the two groups (*p* = 0.15, Figure 2C), however, the DI_OGTT_ was approximately 50% lower among those with a history of CM (*p* = 0.04, Figure 2D).

The CM score was negatively associated with the Matsuda index and positively associated with the HOMA-IR after controlling for age, sex, race, and education (both *p* < 0.05). As child abuse and neglect have great risk for depression, and depressive symptoms were more prominent in the CM group (Table 1), depression may have impacted the measured relationships. Therefore, the associations of Matsuda index and HOMA-IR with CM score were repeated after adding depressive symptoms measured by the BDI as a covariate and the associations remained (*p* = 0.03 and 0.007, respectively). The CM score was not associated with fasting glucose levels, whereas it was associated with fasting insulin levels (*p* = 0.02). In regard to beta cell function, CM score was not associated with insulinogenic index, but was negatively associated with DI_OGTT_ after controlling for age, sex, race, and education (*p* = 0.02). Additional adjustment of depressive symptoms attenuated the significant association between CM score and DI_OGTT_ (*p* = 0.06).

### Associations between Inflammatory Markers and CM

**Figure 3:**
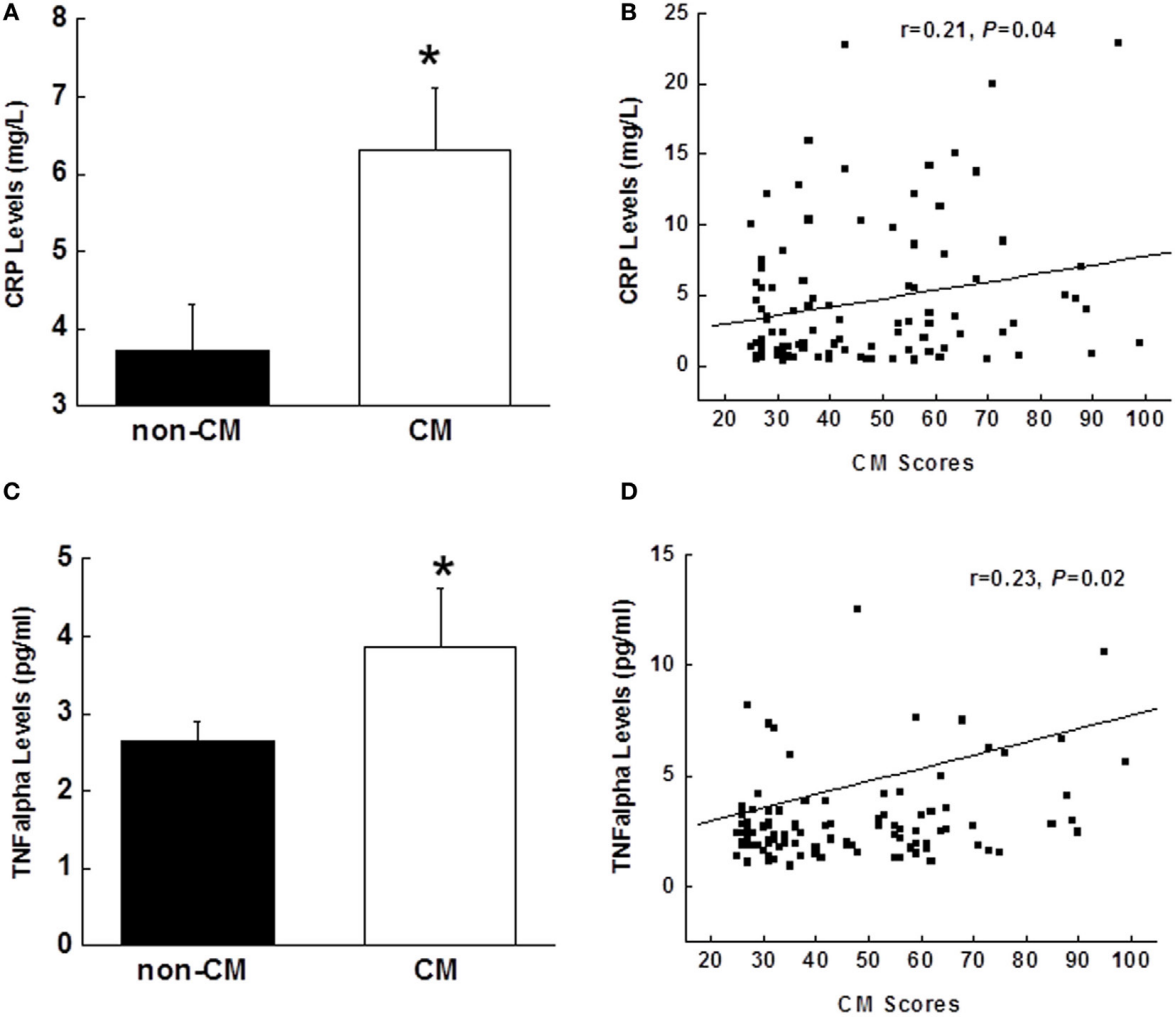
Comparison of plasma concentrations of CRP **(A)** and TNFα **(C)** between the CM and non-CM groups. Partial correlational analysis between CRP and CM scores in **(B)** and between TNFα and CM scores in **(D)**, after controlling for age, gender, race, education, and depression. Data are presented as the mean ± SE in each group. *p < 0.05; compared with the non-CM group. CM, childhood maltreatment; CRP, C-reactive protein; TNFα, tumor necrosis factor-α.

Elevated levels of CRP and TNFα were found in the CM group vs. the non-CM (Figures 3A,C). Pearson partial correlational analysis revealed that there were significant associations between CM score and CRP (*r* = 0.21, *p* = 0.04), and TNFα (*r* = 0.23, *p* = 0.02), after adjusted by age, gender, race, education, and depression symptoms (Figures 3B,D).

### Predictors for Prediabetic State

Compared with the non-CM group, visceral fat mass was significantly greater in the CM group (940 ± 86 vs. 1,202 ± 118, *p* = 0.037). Thus, visceral fat mass, but not BMI and waist-to-hip ratio, was entered into stepwise multiple regression analyses. To determine which risk factors for prediabetic state, including age, race, gender, CM score, depression score, visceral fat mass, TNFα, and CRP, were most predictive of metabolic abnormalities, stepwise multiple regression analyses were performed with these risk factors as the independent variables, and glucose area under the OGTT curve, HOMA-IR, and DI_OGTT_ as the dependent variables in the models. Analyses revealed that only CM entered the regression equation with statistical significance and had independent effects. The model explained 13% of the variance in glucose area under the OGTT curve, and CM contributed significantly (β = 0.35, *p* = 0.013). The model explained 18% of the variance in the HOMA-IR. Significant contributor was CM (β = 0.42, *p* = 0.004). The model explained 11% of the variance in DI_OGTT_ and only CM contributed significantly (β = −0.34, *p* = 0.025).

## Discussion

The current study examined prediabetic state risk in relation to CM, and provided evidence supporting a link between CM and the risk of developing prediabetic state and the subsequent T2D in adulthood. In the present study, we found that 60 min glucose levels and glucose AUC during an OGTT were greater in individuals with CM exposure than in the non-CM group. Insulin levels were not different between the two groups. In the CM group, reduced insulin sensitivity (measured by the Matsuda index and HOMA-IR), independent of age, sex, race, education, and depression, was observed. In line with reduced insulin sensitivity, the disposition index measured using DI_OGTT_ was lower in the CM group. To explore the underlying mechanism for the pathway from the CM to prediabetic state risk, TNFα and CRP levels were measured, and both were significantly elevated in the CM group. Positive relationships were found between both TNFα and CRP levels and CM scores. Stepwise multiple regression analyses revealed that CM was an independent contributor to variance in glucose area under the OGTT curve, HOMA-IR, and DI_OGTT_. Taken together, these results suggest that CM is an independent risk factor for prediabetic state because its association with metabolic parameters was independent of depression. In addition, our findings also indicate that CM plays a critical role in glucose homeostasis and insulin sensitivity in humans.

Since CM has a sustaining negative impact on the health problems, including prediabetic state and T2D, it is essential from a preventative and therapeutic perspective to learn its role. Our findings that individuals with CM experiences have impaired glucose tolerance (i.e., elevated glucose AUC during the OGTT), reduced insulin sensitivity, and impaired beta cell function would suggest a heightened risk for the development of future T2D. In addition, 60 min glucose levels during the OGTT were significantly higher in the CM group and correlated with OGTT-derived Matsuda index (data not shown). It is known that impaired glucose tolerance and reduced insulin sensitivity are present long before the onset of overt T2D (4, 30). Before the conversion of prediabetes to T2D, addressing any potentially modifiable risk factors such as CM and its maladaptive behaviors is therefore relevant in order to reduce the rate of T2D. To date, efforts to prevent T2D have mainly focused on behavioral modifications, such as promoting physical activity and adopting healthy eating habits (36, 37). However, these interventions are often either not realized or achieved with difficulty. The findings of our study may improve public awareness and efforts by identifying the adverse childhood experiences that underlie the more obvious behavioral risk factors. Thus, early recognition, prevention, and treatment of CM may provide an important avenue for the prevention of developing future T2D.

We attempt to explore the mechanisms underlying pathways from CM to glucose intolerance and reduced insulin sensitivity because it allows for the development of more specific targets for the subsequent T2D. Previous studies indicate that childhood stress increases the tendencies for pro-inflammatory status, leading to exaggerated cytokine response to stress and decreased sensitivity to inhibitory hormonal signals and hormonal dysregulation (26, 27). In this study, both TNFα and CRP were found to be elevated and positively associated with CM scores. Greater TNFα and CRP levels in the CM group could contribute to the study findings because both TNFα and CRP have been indicated to promote insulin resistance (38). However, regression analysis did not support that these two inflammatory markers were independently associated with the HOMA-IR and DI_OGTT_, indicating that elevated TNFα and CRP may play a role by mediating other risk factors for T2D such as obesity. Given the nature of our research as a cross-sectional study, it is premature to draw any conclusions. Therefore, prospective studies are warranted to explore this complex relationship.

Previous studies have indicated that obesity is highly related to T2D (17, 18, 35). It is known that risk factors of developing T2D include age, race, gender, and education (39). Therefore, all of these variables including visceral fat mass were treated as independent variables in stepwise multiple regression analyses. Interestingly, all other variables were excluded by regression analyses except CM score in the models. Thus, CM seems to be an independent factor leading to prediabetes manifested by its contribution to glucose intolerance, reduced insulin sensitivity (i.e., HOMA-IR), and reduced DI_OGTT_. We have reservations to generalize our findings; however, appropriate early intervention in glycemic control and improvement in insulin sensitivity in the CM individuals may reduce the rate of prediabetes and T2D.

Our findings have some clinical implications. Due to significant high prevalence of CM and its impact on insulin sensitivity and glucose homeostasis, practitioners should assess for child abuse and neglect history with every patient. Furthermore, clinicians could educate their patients to minimize risky health behaviors and increase stress management techniques to reduce the risk of developing prediabetes and future T2D in individuals who were exposed to childhood adversity. In addition, CM could also be related to problematic diabetes outcomes (e.g., worse adherence, higher HbA1c values) (40). Very few studies to date have examined specific diabetes outcomes in relation to child abuse and neglect. Therefore, our study, along with others, provides the foundation for future research, particularly longitudinal studies, to develop more specific targets for intervention, including family and individual psychotherapy and frequent clinic-based contact, in diabetic patients with CM exposure for better health outcomes. Understanding the association between CM and the risk for prediabetes and T2D may aid in prevention and intervention efforts, ultimately reducing the high rates of mortality and morbidity associated with diabetes.

Several limitations to this study may need to be considered when interpreting our results. First, this study was limited due to the cross-sectional methodology, leading to some reservations regarding its generalizability. However, our findings offered substantial evidence of a strong association between CM and prediabetic state risk in adulthood. Second, underreporting of adverse childhood experiences in our participants probably occurred. Due to the sensitive nature of questions about CM, the responses may represent an underreporting of actual occurrence as a result of being reluctant to report adverse experiences. Third, some health behavior data, including smoking status, diet, and physical activity, might affect our observations. Future studies should collect these data and determine their effects on the health outcomes in individuals with CM. Finally, inconsistency was reported regarding the relationship between subscales of CM (including abuse or neglect) and odds of diabetes, whereas most studies reported a positive association. In the present study, the impact of subscales of CM on prediabetic state risk was unable to be examined due to sample size. Despite the limitations of current study, CM appears to be a construct-relevant factor for prediabetes prevention and intervention.

Understanding risk factors related to the development of prediabetic state and future T2D as well as subsequent poor glycemic control is critical, and CM may be one such risk factor. Our study suggests that CM is independently associated with prediabetic state risk, possibly by impairing glucose tolerance, reducing insulin sensitivity, and beta cell function. This chain of events might begin with childhood exposure to abuse and/or neglect, leading to the development of prediabetes and subsequent T2D as a result of long-term effects of pathophysiological response to the CM. Further study is warranted to address the mediators and moderators, and underlying mechanisms for the pathways from CM to prediabetic state and the subsequent T2D, in the hope of developing specific targets, i.e., CM, to minimize the rate of prediabetic state and T2D and to improve its outcomes.

## Ethics Statement

The project was approved by the Institutional Review Board at the University of Alabama at Birmingham. Written informed consent was obtained from each participant prior to completing any research procedures.

## Author Contributions

LL designed the experiments, analyzed the data, wrote and edited the manuscript. WG contributed to experimental design and the discussion, and reviewed/edited the manuscript. BG contributed to review and edit the manuscript. All authors approved the submission and subsequent publication.

## Conflict of Interest Statement

The authors declare that the research was conducted in the absence of any commercial or financial relationships that could be construed as a potential conflict of interest.

## Abbreviations

AUC, area under the curve; BMI, body mass index; CM, childhood maltreatment; CRP, C-reactive protein; CTQ, Childhood Trauma Questionnaire; DI_OGTT_, OGTT-derived disposition index; HOMA-IR, homeostasis model assessment of insulin resistance; OGTT, oral glucose tolerance test; TNFα, tumor necrosis factor-α; T2D, type 2 diabetes.
